# Phage anti-defense genes targeting NAD^+^ metabolism and DNA replication are associated with a broad host range against *Klebsiella pneumoniae*

**DOI:** 10.1080/21505594.2026.2709261

**Published:** 2026-08-24

**Authors:** Bingbing Lei, Yueren Xu, Keyu Zhu, Kexuan Gao, Yuhui Li, Xia Li, Xinyao Xu, Hui Li, Ruirui Hu, Yue Wang, Jihong Dai, Zhenghao Li, Yuanmeng Lv, Wenjing Ge, Wei Ni, Shengwei Hu

**Affiliations:** a College of Life Sciences, Shihezi University, Shihezi, Xinjiang, China; b Institute of Agro-Product Processing, Xinjiang Academy of Agricultural and Reclamation Science, Xinjiang, Shihezi, China; c Department of Opthalmic Center, Xinjiang 474 Hospital, Xinjiang, Urumqi, China; d Institute of Crop Research, Xinjiang Academy of Agricultural Sciences, Urumqi, China

**Keywords:** Broad-host-range phage, phage therapy, phage anti-defense genes, Klebsiella pneumoniae

## Abstract

Phage therapy for drug-resistant *Klebsiella pneumoniae* (*K. pneumoniae*) is limited by narrow host ranges. We isolated a broad-host-range phage, vB_KP_P4, that lyses 109 diverse *K. pneumoniae* strains, including multidrug-resistant (MDR) and carbapenem-resistant isolates. We suggest that its broad lytic spectrum is likely linked to its capacity to overcome host intracellular defenses. We provide genetic and comparative genomic insights indicating that three phage-encoded genes, deoxycytidylate deaminase (*comEB*), nicotinamide-nucleotide adenylyltransferase (*nadM*), and a DNA polymerase clamp loader (*rfcS*), are correlated with the ability of vB_KP_P4 to infect diverse *K. pneumoniae*. Deletion of these genes collapsed the host spectrum from 109 strains to fewer than 10, significantly compromising antibacterial efficacy. The wild-type phage substantially improves survival in a mouse bacteremia model. Our study identifies candidate genetic factors associated with the broad host range phenotype, specifically targeting host immunity, and provides a new strategy for engineering therapeutic phages.

## Graphical abstract

**Figure:**
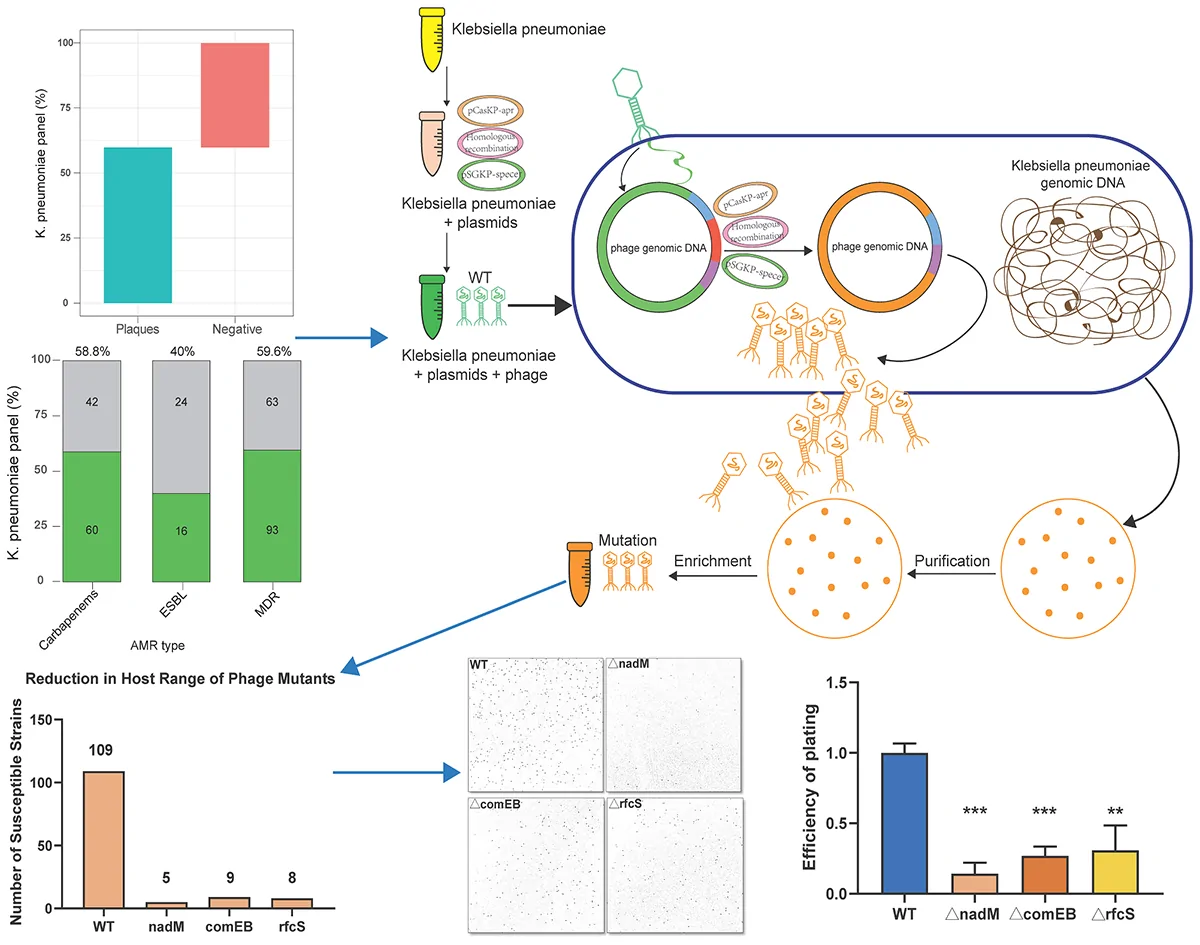

## Introduction

The global public health threat posed by *Klebsiella pneumoniae* (*K. pneumoniae*) has become increasingly serious in recent years, largely due to the overuse of antibiotics and the rapid dissemination of resistant strains [1]. The emergence and dissemination of drug-resistant *K. pneumoniae* in livestock production have made it increasingly challenging to treat bacterial infections in animals. Traditional antibiotic treatment can no longer meet the needs of prevention and treatment of drug-resistant *K. pneumoniae* [2]. Therefore, it is urgent to develop alternative therapies to antibiotics.

Phage therapy, an environmentally friendly alternative to antibiotics, has demonstrated significant efficacy. This success has led to a surge of interest in phage research [3–9]. Despite the promise of phages in combating infections caused by antibiotic-resistant *K. pneumoniae*, clinical application is hampered by the narrow host range of phages and the significant diversity of *K. pneumoniae* strains [10,11]. To overcome this obstacle, two primary strategies have been employed. One approach involves the development of phage cocktails. These combine multiple phages to expand the breadth of bacterial coverage [12–14]. However, the remarkable diversity of drug-resistant *K. pneumoniae* necessitates extensive efforts in phage isolation and validation. This can hinder the clinical application of this approach. Alternatively, identifying phages with an inherently broader host range presents a more streamlined and promising solution for combating drug-resistant *K. pneumoniae*. Therefore, the search for broad host range phages and the expansion of the *K. pneumoniae* phage library are important solutions to combat drug-resistant *K. pneumoniae*.

Bacteriophages infect bacteria through diverse and complex mechanisms. They engage in intricate interactions with their bacterial hosts. The bacterial preference of any given phage, known as its host range, is a critical determinant of its therapeutic potential. Therefore, exploring the host range mechanism of phages is of paramount importance. Emerging research suggests that the expression of phage receptor binding proteins (RBPs) and their subsequent recognition and binding to host surface receptors are critical factors influencing phage adsorption to host bacteria. These steps ultimately dictate host range [15,16]. However, upon entering the host cell, phage DNA encounters various molecular mechanisms that restrict its activity. These include the prevalent Restriction-Modification (RM) systems [17], sequence-specific CRISPR-Cas adaptive immunity [18] that cleaves phage DNA, and abortive infection (Abi) systems [19] that interrupt phage development to protect the bacterial population. Additionally, defense systems like CBASS, prokaryotic Argonaute (pAgo), SEFIR, and Thoeris [20–22] deplete bacterial NAD^+^ levels. This depletion limits the energy required for phage DNA replication. Bacterial defense systems, by interfering with phage DNA replication and reducing burst size, may represent another crucial factor influencing phage host range.

In light of the aforementioned challenges, this study successfully isolated a broad host range lytic bacteriophage, vB_KP_P4, against *K. pneumoniae*. We characterized vB_KP_P4 using whole-genome sequencing, assessed its lytic activity against diverse *K. pneumoniae* strains, and evaluated its therapeutic efficacy and safety in vivo. Linear regression analysis and gene editing techniques were employed to identify key genes influencing the host range of phage vB_KP_P4. This study expands the repertoire of *K. pneumoniae* phages and elucidates the key genes influencing the host range of vB_KP_P4. Our findings provide valuable data for understanding phage host adaptation mechanisms and mining phage anti-defense genes. This research also offers crucial support for developing phage-based therapeutics against antibiotic-resistant *K. pneumoniae* and their clinical application.

## Result

### Isolation and host range of *K. pneumoniae* phage

**Figure 1.**
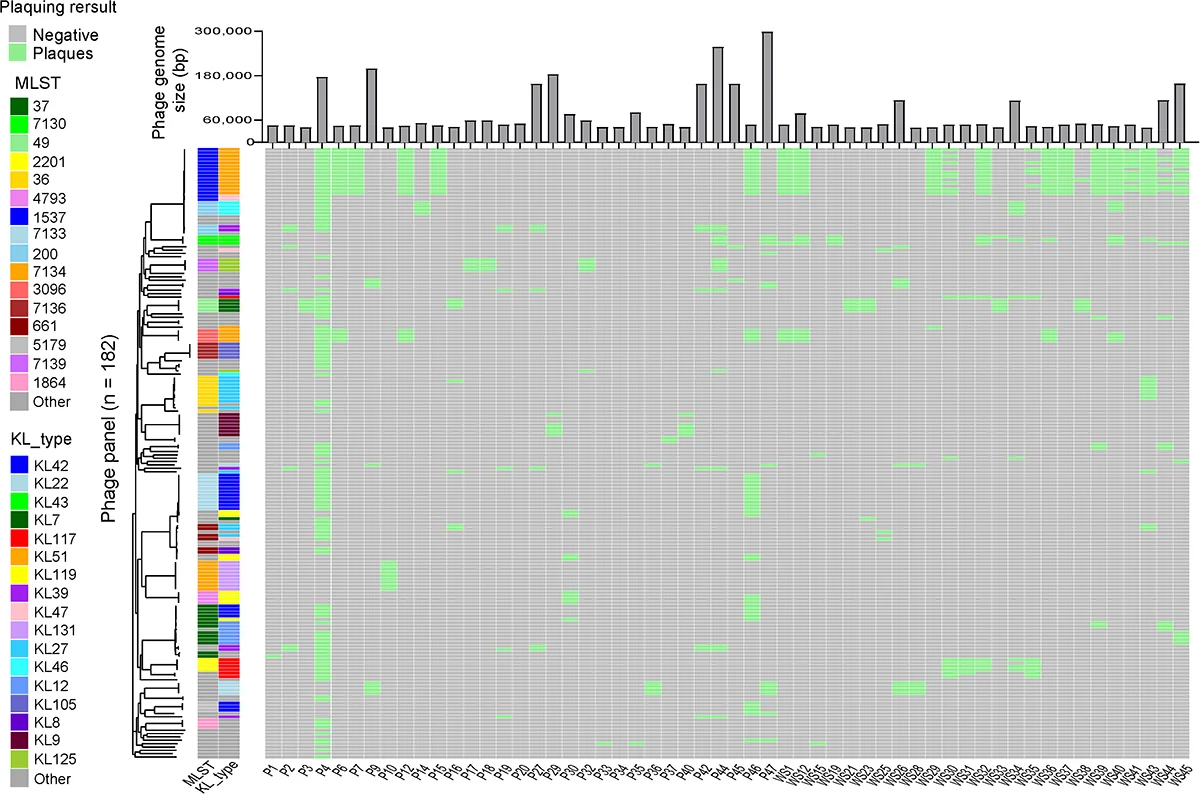
Host range profiles and genome sizes of 56 bacteriophage isolates. Phage susceptibility was assessed using the spot assay, with the presence or absence of plaques after the spot assay indicating *K. pneumoniae* bacterial susceptibility to the phage. Clustering was performed based on the phage host range. Green squares represent bacterial susceptibility to the phage, while gray squares represent resistance. A bar graph above the heatmap displays the genome size of each phage. The phylogenetic tree of *K. pneumoniae* bacteria is shown on the left side of the heatmap, with MLST and KL_type representing two different typing categories for each *K. pneumoniae* bacterium.

In this study, 56 phages were isolated and subjected to host range determination and whole-genome sequencing (Figure 1). The host range of the 56 phages was determined against 182 bovine *K. pneumoniae* strains using the spot test method. The lysis rates of 56 phages against *K. pneumoniae* ranged from 0% to 59.89% (Supplementary Table S1). Among the isolated phages, vB_KP_P4 exhibited a broad host range, capable of lysing 109 bovine-derived *K. pneumoniae* strains, representing a lysis rate of 59.89%. Subsequently, vB_KP_P46 demonstrated lytic activity against 46 strains, with a lysis rate of 25.27%. Notably, both vB_KP_P4 and vB_KP_P46, originating from hospital sewage, effectively lysed *K. pneumoniae* strains from diverse geographical locations. In addition, phages vB_KP_WS43, vB_KP_WS40, vB_KP_WS32, and vB_KP_WS12 exhibited relatively broad host ranges, capable of lysing 25, 22, 22, and 21 *K. pneumoniae* strains, respectively.

### Whole genome analysis of *K. pneumoniae* phage

Whole-genome sequencing of the 56 bacteriophage isolates revealed significant genomic diversity, with genome sizes ranging from 39 kbp to 298 kbp and GC contents spanning 41.8% to 57.1%. Taxonomically, these phages were assigned to nine distinct families, including Autographiviridae and Straboviridae. Six isolates were identified as temperate phages, while the remaining 50 were virulent (Supplementary Figure S1). Detailed genomic metrics and taxonomic classifications are summarized in Supplementary Tables S2 and S3.

### Evolutionary characteristics of *K. pneumoniae* phage

**Figure 2.**
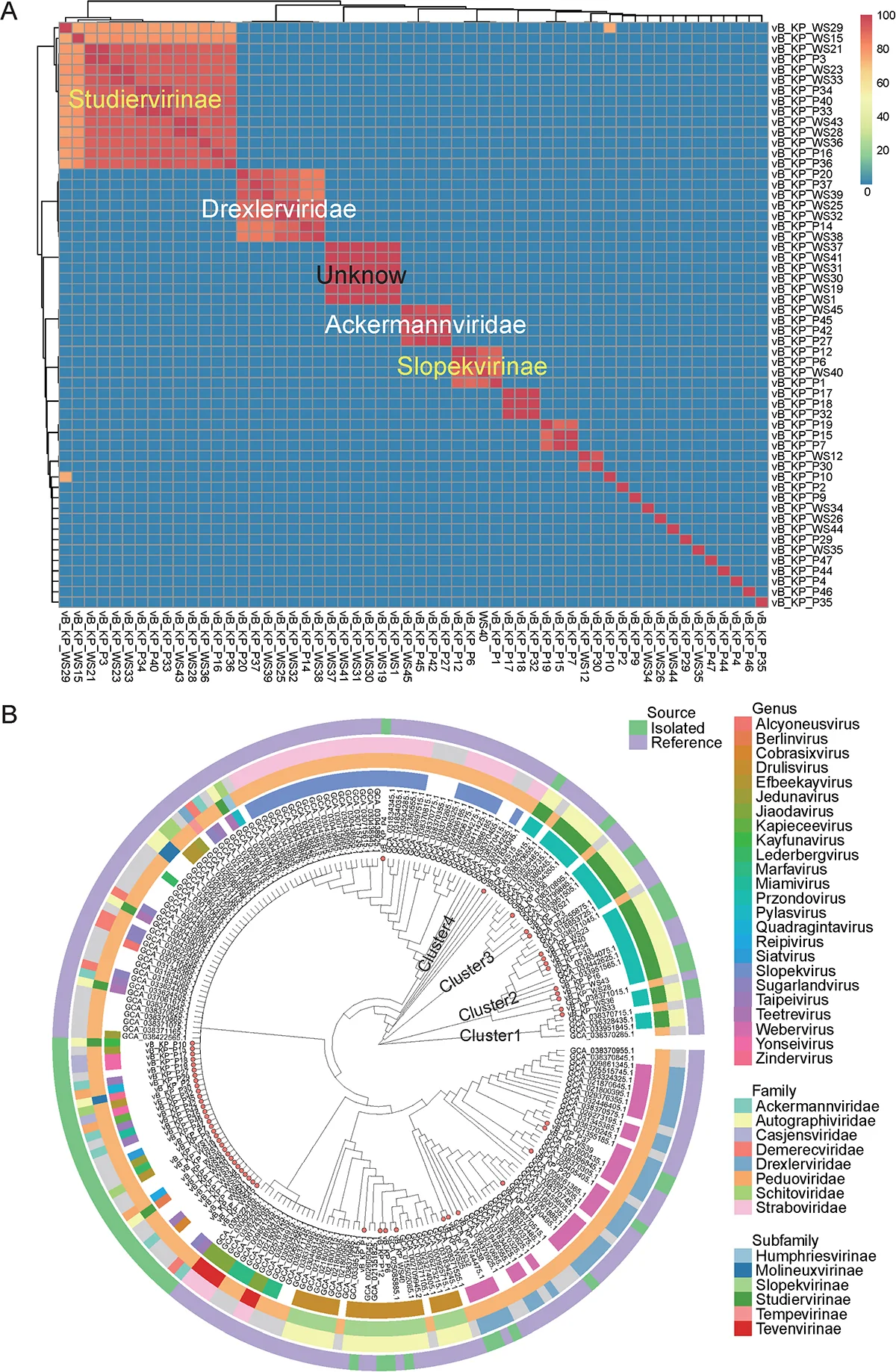
Evolutionary characterization of 56 *K. pneumoniae* phages based on whole-genome sequencing. A. Depicts an ANI heatmap of 56 phages. Phages are represented along both axes, with color intensity indicating the ANI values between phages. B. A phylogenetic tree of 56 phages isolated in this study and 160 *K.* *pneumoniaes* phages from the NCBI RefSeq database. Red circles at the branch tips represent phages isolated in this study, while unlabeled ones are from the NCBI database. The outer bands of the phylogenetic tree (from inner to outer) represent the classification of phages at the genus, subfamily, and family levels, respectively, with the outermost band representing the source classification of the phages.

To elucidate the phylogenetic relationships among the bacteriophages, a whole-genome phylogenetic tree was constructed for the 56 bacteriophages included in this study, and the Average Nucleotide Identity (ANI) was calculated for each phage (Figure 2(A)). The analysis revealed that the ANI values among the phages ranged from 0.746 to 0.999. Furthermore, five distinct clusters were observed, corresponding to two families (Drexlerviridae and Ackermannviridae), two subfamilies (Studiervirinae and Slopekvirinae), and one unclassified group, with each cluster comprising closely related phages (ANI >70%). To further investigate the phylogenetic relationships among the 56 bacteriophages isolated in this study, a whole-genome-based phylogenetic tree was constructed, incorporating the genomes of 160 additional bacteriophages downloaded from NCBI. The resulting phylogenetic analysis, as depicted in Figure 2(B), revealed that the 216 bacteriophages formed four distinct clades. Specifically, clade 1 comprised 5 phages, clade 2 contained 15 phages, and clade 3 harbored 9 phages. Notably, all phages within these three clades belonged exclusively to the genus Przondovirus. In contrast, clade 4 encompassed 187 phages, representing 23 different genera.

### Characteristic analysis of the broad host range phage vB_KP_P4

**Figure 3.**
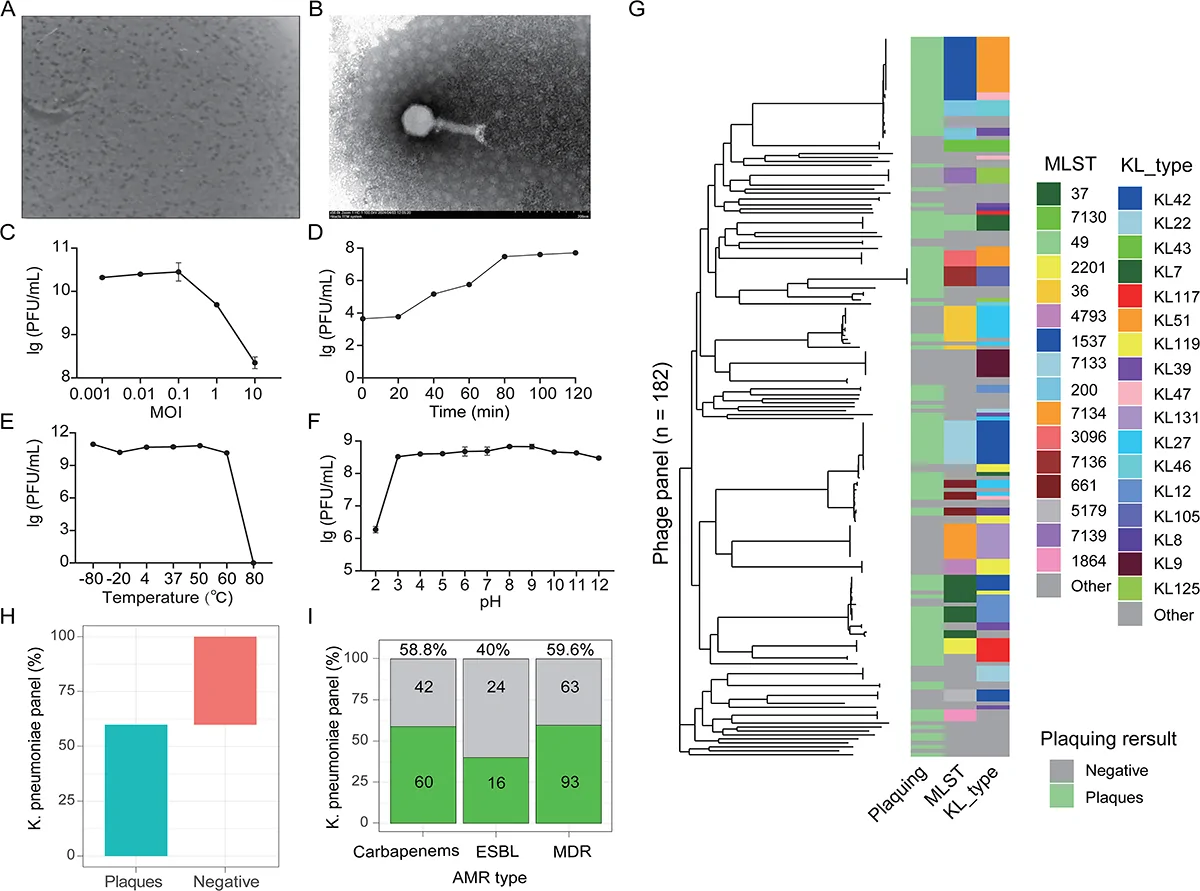
Characteristic analysis of bacteriophage vB_KP_P4. A. Plaque morphology of phage vB_KP_P4. B. TEM of phage vB_KP_P4. C-F. Multiplicity of infection (MOI); one-step growth curve; temperature stability; pH stability. The entire experiment was independently repeated three times. G. Unrooted phylogenetic tree of *K. pneumoniae* strains, showing 182 *K. pneumoniae* strains, encompassing 67 MLST types and 48 *K. pneumoniae* KL_types. Plaque assay data showing the lytic activity of phage vB_KP_P4 against *K. pneumoniae* strains, with green squares indicating lysis by vB_KP_P4 and gray squares indicating no lysis. H. Plaque assay used to analyze the lysis rate of vB_KP_P4 against 182 KP strains. I. vB_KP_P4 lyses more than 50% of carbapenem-resistant and multidrug-resistant *K. pneumoniae* strains, and 40% of extended-spectrum beta-lactamase (ESBL) resistant *K. pneumoniae* strains. Numbers displayed in each green or gray bar represent the number of *K. pneumoniae* strains sensitive or resistant to vB_KP_P4, respectively, from the 182 strains screened.

The unique characteristics of bacteriophage vB_KP_P4 were evident across host range determination, ANI analysis, and phylogenetic tree analysis, prompting a comprehensive characterization of its properties. Bacteriophage vB_KP_P4 formed small, pinpoint-sized plaques without halos (Figure 3(A)). Transmission electron microscopy (TEM) revealed that vB_KP_P4 possesses an icosahedral head and a contractile tail (Figure 3(B)). The optimal multiplicity of infection (MOI) was determined to be 0.1, with a latent period of 20 minutes. The phage exhibited a burst phase from 20 to 80 minutes, reaching a burst size of 54 PFU/cell, followed by a plateau phase (Figure 3(C,D)).  Bacteriophage vB_KP_P4 remained stable across temperatures from −80°C to 60°C, showing minimal fluctuations in activity. In contrast, vB_KP_P4 was completely inactivated after 1 hour of exposure to 80°C (Figure 3(E)). The phage demonstrated robust environmental adaptability, maintaining stable titers after 1 hour of exposure to pH conditions ranging from 3.0 to 12.0. However, asignificant reduction in phage activity was observed at pH 2.0 (Figure 3(F)).

To evaluate the lytic activity of bacteriophage vB_KP_P4 against *K. pneumoniae*, its coverage was tested against 182 bovine-derived *K. pneumoniae* isolates. The lytic spectrum of vB_KP_P4 against *K. pneumoniae* is presented in Figure 3(G). The host range of vB_KP_P4 covered 58.21% (39/67) of molecular types and 64.58% (31/48) of serotypes. As shown in Figure 3(H), the overall lytic rate of vB_KP_P4 against the 182 *K. pneumoniae* isolates was 59.89% (*n* = 109). Specifically, vB_KP_P4 lysed 59.6% (*n* = 93) of MDR strains, 58.8% (*n* = 60) of carbapenem-resistant strains, and 40% (*n* = 16) of extended-spectrum β-lactamase (ESBL) resistant strains (Figure 3(I)).

### Genomic characteristics of bacteriophage vB_KP_P4

**Figure 4.**
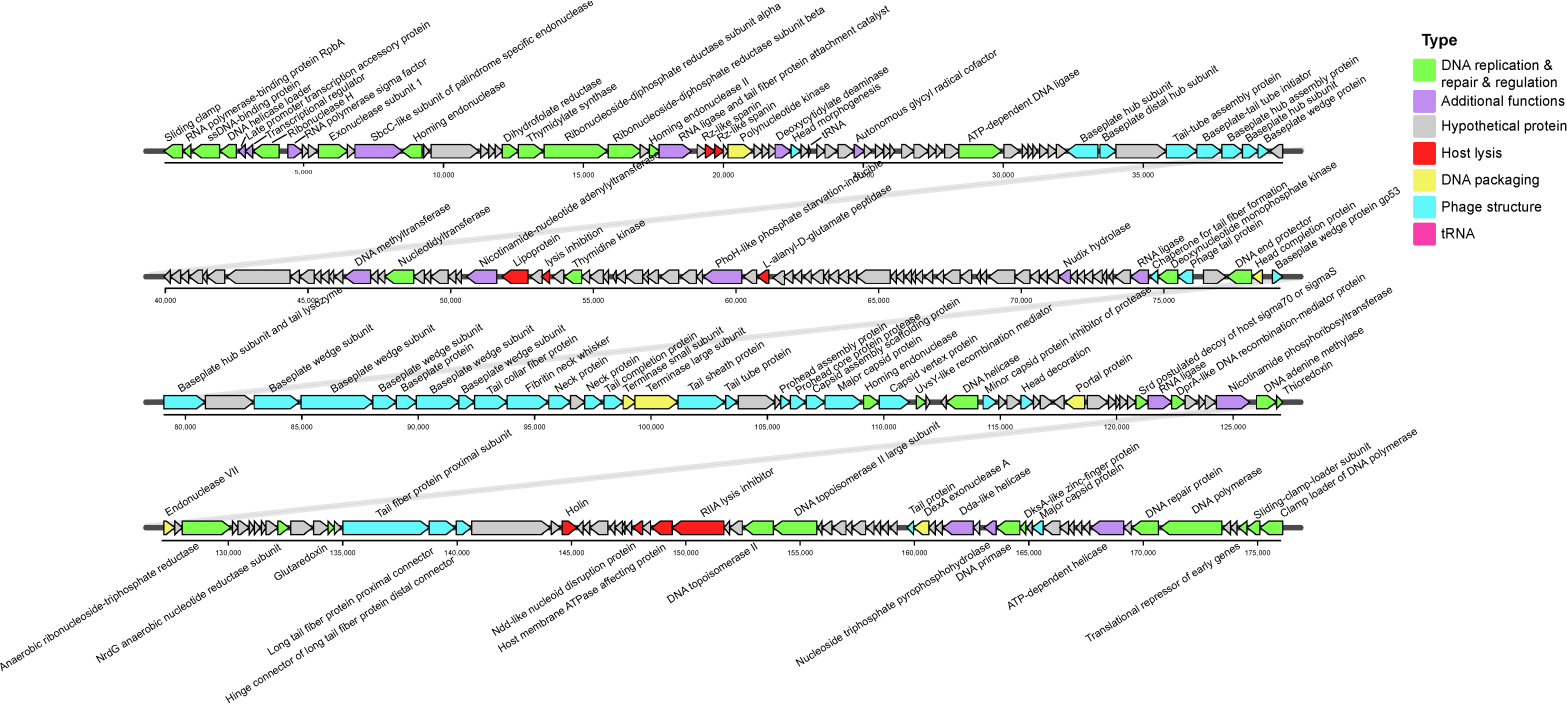
Genetic linear structure of bacteriophage vB_KP_P4. Arrows of varying colors denote distinct categories of genes.

The complete genome of bacteriophage vB_KP_P4 is 176,180 bp in length, with a GC content of 41.8%, and consists of double-stranded linear DNA. Based on its morphology and genomic analysis, it was classified into the family Straboviridae. Genome annotation using Prokka revealed that vB_KP_P4 encodes 1 tRNA and 272 protein-coding genes. A BLASTp homology search of the 272 coding sequences identified 106 genes with specific functional annotations (Supplementary Table S4), while 166 genes lacked discernible homology to sequences with known functions and were annotated as hypothetical proteins. Based on their characteristics and encoded proteins, the 106 functionally annotated genes were categorized into five classes: DNA replication & repair & regulation, phage structure, DNA packaging, host lysis, and additional functions, as detailed in Supplementary Table S5. Notably, we identified several genes potentially involved in bypassing host immunity, including a nicotinamide-nucleotide adenylyltransferase (*nadM*, ORF_091) and a deoxycytidylate deaminase (*comEB*, ORF_035). These candidates were prioritized for subsequent knockout studies to investigate their role in host range determination. The linear gene organization of bacteriophage vB_KP_P4, depicted in Figure 4, was generated using Easyfig software.

The absence of any genes related to virulence, antibiotic resistance, or lysogeny in bacteriophage vB_KP_P4 indicates that it will neither transfer resistance nor virulence determinants to bacteria nor integrate into the bacterial genome during its propagation. This genomic analysis provides evidence for the safety of vB_KP_P4, suggesting its potential applicability as a therapeutic agent. The basic biological information of bacteriophage vB_KP_P4 is summarized in Supplementary Table S2.

### Factors affecting the host range of vB_KP_P4

**Figure 5.**
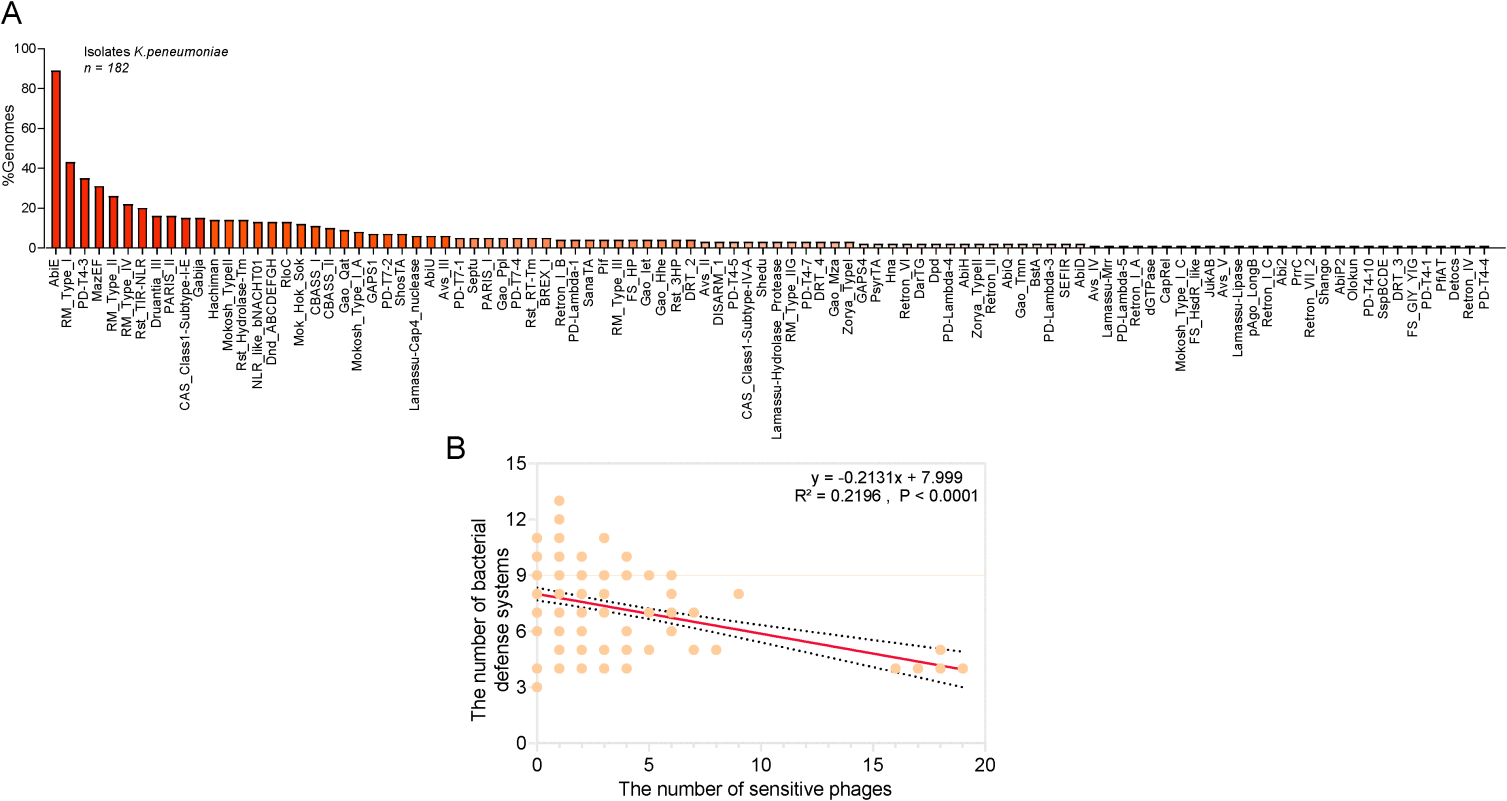
Correlation analysis between the accumulation of bacterial defense systems and the host range of phages. A. Diversity of bacterial defense systems identified in *K. pneumoniae* isolate genomes, ranging from most abundant (left) to least abundant (right). Supplementary Table S6 is a comprehensive list of defense systems. B. Linear regression analysis of the correlation between the number of bacterial defense systems identified in *K. pneumoniae* genomes and the number of susceptible phages. R^2^ represents R-squared, a goodness-of-fit measure for the linear regression models.

During the co-evolution of bacteria and bacteriophages, bacteria continually evolve defense mechanisms to counteract phage infection. This study aimed to explore the correlation between the accumulation of bacterial defense systems and phage resistance by predicting defense mechanisms in *K. pneumoniae* isolates. As depicted in Figure 5(A), the 182 *K. pneumoniae* isolates harbored a diverse repertoire of approximately 100 defense mechanisms, including RM, CBASS, Abi, AVAST, SEFIR, Retron, Lamassu-Fam, Zorya, BREX, pAgo, and PARIS, indicating a rich diversity of defense mechanisms in *K. pneumoniae*. Supplementary Figure S2 revealed that each *K. pneumoniae* isolate contained at least three defense systems. Correlation analysis between the number of defense systems and the number of susceptible phages (Figure 5(B)) demonstrated a negative correlation (R^2^ = 0.2196, *p *< 0.0001), suggesting that an increased number of defense systems in *K. pneumoniae* isolates is associated with a decreased number of susceptible phages.

RM, CBASS, AVAST, SEFIR, BREX, pAgo, and PARP are all bacterial defense systems involved in modulating phage DNA replication within host cells. These systems may limit the host range of phages by inhibiting the DNA replication of phages. In the genomes of 182 bovine *K. pneumoniae* strains in this study, the defense system subtypes that affect phage DNA replication accounted for the largest proportion, accounting for 16% of the total defense system subtypes. To investigate the genetic determinants of lytic breadth, three candidate genes (ORF_035, ORF_091, and ORF_273) were selected for targeted deletion based on their functional annotations.

**Figure 6.**
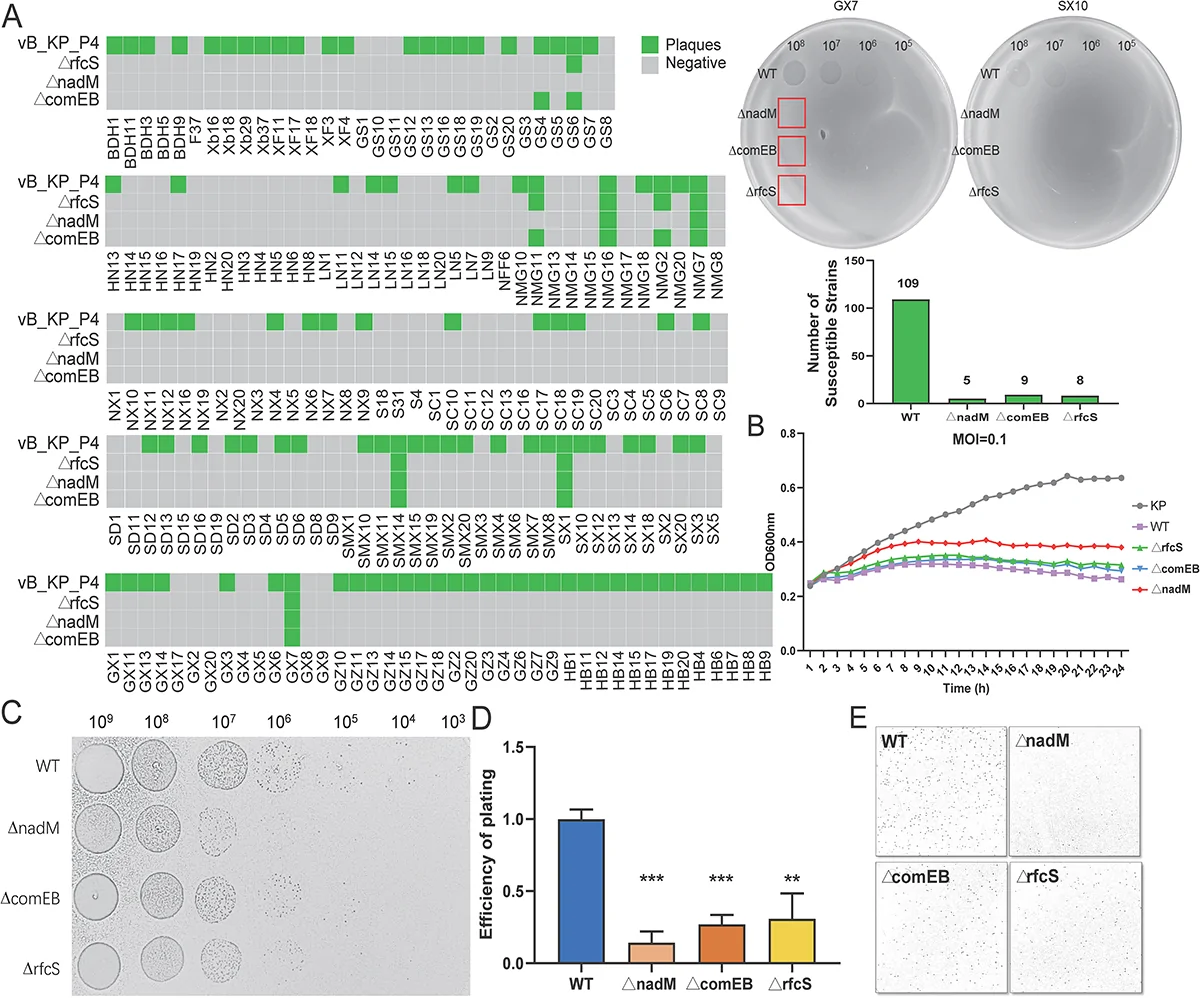
Characterization of mutant phage host range, antibacterial activity, and replication capacity. A. Host range of wild-type (WT) vB_KP_P4 and its mutants against 182 *K. pneumoniae* strains. The heatmap (left) displays the qualitative lytic profile (green square, susceptible; gray squares, resistant), highlighting nuanced activities such as the slight lysis of strain GX7 by the mutants, while strain SX10 became fully resistant. The bar chart (right) quantifies the total number of susceptible strains, showing a drastic reduction in host range from 109 for WT to 5 (∆*nadM*), 9 (∆*comEB*), and *8 (∆rfcS*) for the respective mutants. B. In vitro antibacterial activity of vB_KP_P4 and its mutants was determined. C-E. Lytic efficiency and plaque morphology of WT and mutant phages. C. Spot assays of 10-fold serial dilutions of phage lysates. D. Efficiency of plating (EOP) calculated relative to the WT phage (EOP = 1.0). The EOP for ∆*nadM*, ∆*comEB*, and ∆*rfcS* was significantly reduced to 0.142, 0.269, and 0.308, respectively. The entire experiment was independently repeated three times. E. Plaque morphology observed in a double-layer agar assay. Both assays visually demonstrate the severely impaired lytic activity and reduced plaque-forming ability of the mutant phages compared to the WT. In B-E, *K. pneumoniae* isolate NMG16 was used as the host strain.

Compared to the wild-type bacteriophage vB_KP_P4, the host range of the mutant phages ∆*nadM*, ∆*comEB*, and ∆*rfcS* was significantly narrowed, decreasing from 109 host strains to 5, 9, and 8, respectively. And it was observed that the phage mutants were only slightly lytic against some strains (Figure 6(A)), indicating that the genes *nadM*, *comEB*, and *rfcS* influence the host range of vB_KP_P4. The bacteriostatic results, as shown in Figure 6(B), revealed that the inhibitory capacity of the mutant phages was lower than that of the wild-type phage. The lytic efficiency of a phage against susceptible strains reflects its ability to lyse host bacteria. Lytic efficiency was determined using spot assays and double-layer agar plate assays, and the efficiency of plaque formation (EOP) was calculated. The results showed that, with equal density and volume of phage application in the spot assay, the number of plaques formed by the mutant phages was lower than that formed by the wild-type phage (Figure 6(C)), consistent with the results from the double-layer agar plate assay (Figure 6(E)). Taking the EOP of the wild-type phage as a reference (1.0), the EOP of the mutants ∆*nadM*, ∆*comEB*, and ∆*rfcS* was significantly reduced to 0.142, 0.269, and 0.308, respectively (Figure 6(D)). Collectively, these findings demonstrate that the genes *nadM*, *comEB*, and *rfcS* have a substantial impact on the host range, bacteriostatic activity, and lytic efficiency of bacteriophage vB_KP_P4.

In this study, the genomic architecture was analyzed using BPROM (Supplementary Figure S3). Independent, high-scoring promoters were identified upstream of each target gene, including *nadM* (LDF score = 6.54) and *rfcS* (LDF score = 1.13), indicating potential transcriptional autonomy. Furthermore, substantial intergenic regions (e.g. 176 bp preceding *nadM*) reduce the likelihood of transcriptional interference, supporting the functional independence of each locus. However, despite these bioinformatic predictions, the possibility of polar effects or secondary genomic compensation cannot be definitively excluded in the absence of complementation experiments.

Given that the deletion of this triad significantly compromised the lytic efficiency of vB_KP_P4, we explored whether these loci constitute a conserved “anti-defense module” prevalent among wide-spectrum phages. To test this hypothesis, we extended our analysis to evaluate the universality of these genes beyond vB_KP_P4. We performed a comparative genomic meta-analysis of 20 previously characterized *K. pneumoniae* phages with documented host-range profiles (Supplementary Table S7). Statistical validation using Fisher’s exact test revealed a significant correlation between the presence of this gene cluster and the broad host range phenotype (*p* = 0.0002; Supplementary Figure S4A). This robust correlation stems from the observation that 100% (16/16) of broad host range phages naturally harbored this complete module, whereas it was entirely absent in the narrow-spectrum counterparts. Notably, the module was exclusively enriched in lineages exhibiting superior lytic breadth (lysis rates > 20%), while being completely missing from restricted narrow-spectrum genomes. Furthermore, the high sequence conservation (>98% identity) of these functional genes between vB_KP_P4 and well-characterized clinical phages (e.g. phiKp8) suggests that these genes may contribute to overcoming bacterial immunity in diverse phage lineages (Supplementary Figure S4C-E). This striking evolutionary conservation across diverse lineages (Supplementary Figure S4B) reinforces the role of these genes as candidate genomic signatures associated with broad host range in *K. pneumoniae* phages.

### Therapeutic effect of bacteriophage vB_KP_P4

**Figure 7.**
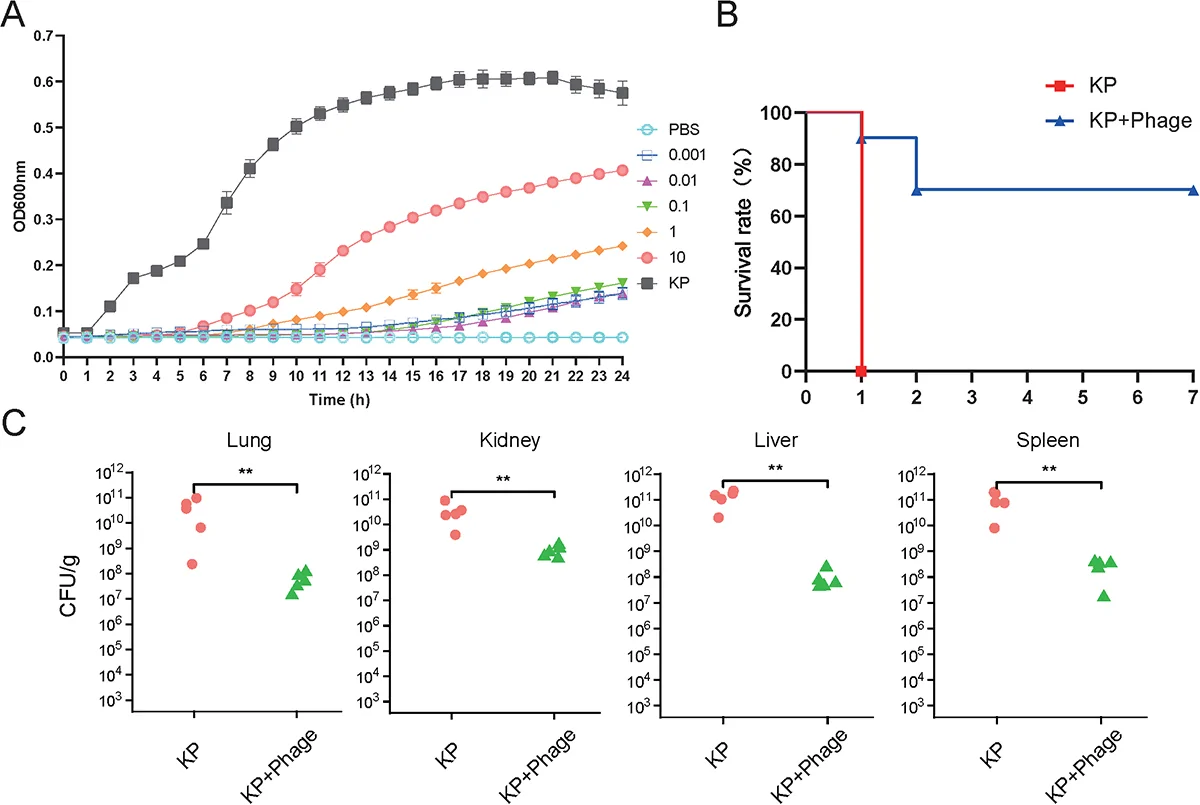
In vivo and in vitro evaluation of bacteriophage vB_KP_P4 in the treatment of *K. pneumoniae* infection. A. in vitro antibacterial assay of vB_KP_P4, using KP isolate NMG16 as the host bacterium, to determine the antibacterial effect of vB_KP_P4 at different MOIs. The negative control was PBS+LB medium, 10–0.001 represents different phage-to-bacteria ratios, and the positive control was *K. pneumoniae*+LB medium. There were four technical repetitions in the experiment. B. Survival rate of *K. pneumoniae*-induced bacteremia model mice treated with vB_KP_P4 (*n* = 10). C. Bacterial burden of *K. pneumoniae* in different tissues (*n* = 5), represented in red (control group) and green (phage-treated group). Each data point represents an individual mouse. Statistical analysis was performed using the Student’s t-test.

In vitro bacteriostatic assays (Figure 7(A)) demonstrated that the absorbance values for all multiplicity of infection (MOI) groups were lower than those of the positive control. Notably, bacterial concentrations increased rapidly at 6 h and 10 h post-infection for MOI of 10 and 1, respectively, resulting in a sharp rise in absorbance. In contrast, bacterial concentrations began to increase at 15 h post-infection for MOI of 0.1, 0.01, and 0.001. This increase in bacterial concentration may be attributed to the emergence of resistant strains. The time to emergence of these resistant strains varied with the MOI, with higher MOI potentially leading to earlier development of resistance.

In vivo experiments demonstrated that bacteriophage vB_KP_P4 significantly enhanced the survival rate of mice infected with *K. pneumoniae* (Figure 7(B)). Bacterial loads in the lung, kidney, liver, and spleen tissues of the mice were quantified. The results revealed that, compared to the *K. pneumoniae*-infected group, the bacterial counts of *K. pneumoniae* were significantly reduced by 2.4 lg10 (*p* < 0.01) in the lung, 1.4 lg10 (*p* < 0.01) in the kidney, 3.2 lg10 (*p* < 0.01) in the liver, and 2.6 lg10 (*p* < 0.01) in the spleen (Figure 7(C)).

## Discussion

The development and continuous evolution of MDR *K. pneumoniae* have led to increasingly limited antibiotic options and poor therapeutic prognoses for associated infections [23], making the development of alternative antibiotic therapies imperative. Among current alternatives, phage therapy has demonstrated significant efficacy. Phage therapy can be broadly categorized into personalized therapy and cocktail therapy. Personalized therapy involves the custom selection of phages tailored to individual patient needs. The high specificity of phages allows for the selective targeting and elimination of pathogenic bacteria, while preserving the patient’s native microbiota [24]. Personalized phage therapy harnesses the high specificity of bacteriophages, necessitating an extensive phage library for optimal therapeutic efficacy. Cocktail phage therapy, requiring pre-configuration and combination to broaden host range, has demonstrated significant efficacy against burn wound infections, murine hepatic injury, and mastitis [25,26], with biphasic cocktails often exhibiting superior therapeutic outcomes compared to monophasic phage therapy [27]. While the comparative efficacy of phage cocktails over monophage therapy requires further investigation, monophage therapy remains compelling due to the substantial resources required for phage identification, development, and purification. In conclusion, the foundation of effective phage therapy lies in an expansive phage library, necessitating the continued expansion of such libraries to achieve optimal therapeutic outcomes.

Of the 56 *K. pneumoniae* bacteriophages in this study, 6 isolates were temperate phages, while the remaining 50 were virulent phages. Furthermore, Average Nucleotide Identity (ANI) and whole-genome phylogenetic analyses revealed significant genomic diversity among the 56 bacteriophages, providing valuable data for expanding the *K. pneumoniae* phage library. In-depth characterization of bacteriophage vB_KP_P4 revealed its strong burst capacity and remarkable environmental stability [28]. This robust stability underscores the phage’s potential for diverse environmental applications. For instance, the normal rumen pH range in cattle is 5.3 to 6.93 [29], maintained at relatively stable levels over extended periods [30]. The observed pH stability suggests that vB_KP_P4 could remain active within the bovine rumen environment.

Bacteriophages infect bacteria by attaching to their surface, and the capsule (K antigen), a crucial bacterial external structure, has been demonstrated to be a primary receptor for many phages [31–35]. In this study, vB_KP_P4 exhibited a broad host range, infecting strains representing 39 molecular types and 31 serotypes, thus confirming its extensive host adaptability. Furthermore, vB_KP_P4 harbors numerous RNA polymerase-related proteins and subunits, indicating that its gene expression may rely solely on its own RNA polymerase, independent of the host’s, consistent with studies by Pieter-Jan and Yihui Yuan et al. [36,37]. Some studies define giant bacteriophages as those with genomes exceeding 200 kbp [38]. These giant phages possess genes absent in smaller genome phages, such as those encoding proteins responsible for genome replication and nucleotide metabolism [39,40]. Notably, some giant phages encode genes that can redirect ATP synthesis to enhance their own propagation within host cells [41]. vB_KP_P4 also encodes nicotinamide nucleotide adenylyltransferase (ORF_091) and nicotinamide phosphoribosyltransferase (ORF_202), two key enzymes in nicotinamide adenine dinucleotide (NAD^+^) biosynthesis. These enzymes are implicated in various bacterial anti-phage defense systems [22,42–44], where NAD^+^ depletion is employed to inhibit phage replication. Conversely, phages possess enzymatic pathways that enable NAD^+^ reconstitution from degradation products within infected cells [45,46]. The identification of *nadM* and *comEB* as key host-range genes suggests that metabolic reconstitution of NAD^+^ is a pivotal strategy for overcoming host suicidal defenses.

While most bacteriophages exhibit a narrow host range, infecting only a subset of strains within a bacterial species [47,48], some phages demonstrate a broader host range, rendering them more suitable for combating bacterial infections or as prophylactic agents [49,50]. In this study, both in vitro and in vivo assays confirmed the potent inhibitory effect of vB_KP_P4 on *K. pneumoniae* growth. The bacteriostatic efficacy of vB_KP_P4 against *K. pneumoniae* varied with different multiplicities of infection (MOIs). Furthermore, we observed a dose-dependent effect of bacteriophage administration on the survival rates of bacteremic mice, which aligns with previous findings [51,52]. Interestingly, some studies have reported superior therapeutic efficacy with low-dose phage treatment compared to high-dose regimens [53]. These discrepancies in therapeutic outcomes across phage dosages highlight the importance of considering phage titer flexibility in phage therapy protocols. Consistent with the prior investigations, phage administration has been demonstrated to significantly reduce bacterial burden in various murine tissues and organs [54].

The host range of a bacteriophage refers to the taxonomic diversity of hosts it can successfully infect, a trait intricately linked to the phage’s infection lifecycle. Current investigations into the molecular mechanisms underlying broad-host-range phages predominantly focus on the adsorption stage [55–57]. Recent studies have shown that host defense systems may prevent phage infection by cleaving the phage genome or inhibiting phage DNA replication, thereby affecting the host range of phages [58–60]. In this study, ORF_035 (*comEB*) encodes deoxycytidylate deaminase, which this enzyme is traditionally associated with bacterial nucleotide metabolism [61]. In the context of phage infection, it likely facilitates the redirection of the host’s nucleotide pool toward phage-specific DNA synthesis, a strategy frequently employed by large dsDNA phages to evade host restriction systems. The gene encoded by ORF_091 (*nadM*) plays a crucial role in NAD^+^ metabolism. Reflecting an emerging paradigm in the phage-host “metabolic arms race,” recent high-impact studies have demonstrated that phages utilize enzymes like *nadM* and *NAMPT* to rewire cellular NAD^+^ pools [45,46]. This mechanism directly counteracts bacterial defense systems, such as CBASS, Thoeris, and Pycsar, which trigger abortive infection by depleting essential NAD^+^ molecules [62,63]. The presence of *nadM* in vB_KP_P4 is consistent with previously described counter-defense mechanisms, providing a plausible biochemical rescue strategy that maintains phage propagation across diverse defense landscapes. Conversely, the clamp loader encoded by ORF_273 (*rfcS*), a critical component of the clamp-loading complex during genome replication, has been shown to significantly impact phage propagation [64]. The host-range contraction of Δ*comEB*, Δ*nadM*, and Δ*rfcS* mutants from 109 to fewer than 10 strains underscores a strong functional link between these genes and lytic breadth. Although BPROM predictions suggest transcriptional autonomy through independent promoters (Supplementary Figure S3), the potential for polar effects cannot be definitively excluded without heterologous gain-of-function assays. Nevertheless, our results in 182 wild-type isolates suggest that these genetic factors significantly contribute to overcoming the integrated defense landscapes encountered in natural infections.

While the host library utilized in this study primarily comprises bovine-derived isolates, the marked evolutionary conservation of *comEB*, *nadM*, and *rfcS* underscores their broad functional significance. The near-identity (>97%) of these functional genes with orthologs in clinically potent phages indicates that the metabolic rescue and replication-enhancement strategies identified in vB_KP_P4 are highly conserved features among phages capable of successfully infecting human clinical pathogens. Consequently, these findings provide a robust molecular framework for understanding phage-mediated evasion of host defenses across diverse ecological niches. Furthermore, these conserved genetic loci serve as predictive molecular markers, enabling genomic-based prioritization of potent phages for personalized clinical interventions.

In conclusion, this study successfully characterized a novel broad-host-range *K. pneumoniae* phage, vB_KP_P4, and validated its therapeutic potential. Crucially, we provide genetic evidence that *nadM*, *comEB*, and *rfcS* are strongly associated with the broad host range phenotype of vB_KP_P4. However, complementation and gain-of-function studies will be required to establish causality and sufficiency. While establishing a direct mechanistic linkage through isogenic strains carrying single, defined defense systems (such as RM or CBASS) remains an essential next step to pinpoint the specific anti-defense functions of these genes, our current findings in wild-type isolates support an important role in overcoming complex bacterial defense landscapes. The “many-versus-many” interactions observed in our diverse isolate panel highlight that these genes likely serve as essential hubs for countering the collective “defense landscape” of MDR *K. pneumoniae*. Consequently, our findings open new avenues for the rational engineering of therapeutic phages, suggesting that these modules may represent promising targets for future host-range engineering efforts.

## Limitations of the study

Despite the clear reduction in host range observed in our deletion mutants, several limitations should be considered. First, technical challenges in manipulating and packaging large dsDNA myoviruses prevented complementation or gain-of-function experiments, such as introducing the *nadM-comEB-rfcS* gene cluster into a narrow host range phage. While potential polar effects cannot be fully excluded, the combined genetic and promoter analyses strongly support the functional association of these genes with the broad host range phenotype, representing functional necessity rather than absolute causality.

Second, although comparative genomic analyses reveal a robust association between the *nadM-comEB-rfcS* module and broad host range, the precise molecular mechanisms remain to be elucidated. Future advances in modular phage assembly and synthetic virology will facilitate direct experimental validation of the sufficiency and mechanistic roles of these candidate genetic modulators.

## Materials and methods

### Chemicals and reagents

Peptone (Cat# LP0042) and yeast extract (Cat# LP0021) were purchased from Thermo Scientific (Waltham, USA). The agar powder (Cat# BS195) was purchased from Biosharp (Hefei, China). The apramycin (Cat# A429656), isoflurane (Cat# A425890), and 4% paraformaldehyde (Cat# E672002) were purchased from Sangon Biotech (Shanghai) Co., Ltd. The MacConkey medium (Cat# HB6238) was purchased from Qingdao High-tech Industrial Park Haibo Biotechnology Co., Ltd.

### Sample origin

*K. pneumoniae* strains were maintained in our laboratory. The collection comprised 182 bovine-derived and 5 human-derived *K. pneumoniae* isolates. The 182 bovine *K. pneumoniae* isolates were utilized to determine the host range of the bacteriophage, encompassing 67 molecular types and 48 serotypes. Samples for phage isolation were collected from sewage at a hospital wastewater treatment plant and domestic sewage from a municipal sewage treatment plant in Shihezi.

This study used 30 female BALB/c mice, 6–8 weeks old and weighing 18–22 g, with 5 mice per cage, purchased from Henan Skebes Biotechnology Co., Ltd (the animal license number is SCXK 2020–0005). All mice were in good condition and were included in the experiment. No animals were excluded during the experiment; all data from the survival study (*n* = 10 mice) and the bacterial load study (*n* = 5 mice) were used for statistical analysis. After the experiment, mice were anesthetized with 3–5% isoflurane and then euthanized by cervical dislocation. All experimental procedures involving animals were conducted in strict accordance with the guidelines and with approval from the Biology Ethics Committee of Shihezi University (approval number A2025-553).

Plasmids pCasKP-apr and pSGKP-km were purchased from Baosai Plasmid and Strain Resource Co were used in these experiments. The synthesis of sgRNAs and the construction of recombinant plasmids required for gene editing were performed by Shanghai Sangon Biotech Co., Ltd.

### Isolation and purification of bacteriophages

Bacteriophages were isolated and purified using the double-layer agar (DLA) method [65]. Briefly, 100 µL of phage dilution was mixed with 100 µL of host bacterial suspension and added to 5 mL of 55°C LB semi-solid agar medium. The mixture was then poured onto a solid LB agar plate and incubated at 37°C for 6 hours. This procedure was repeated 3–5 times until plaques of uniform size and morphology appeared on the agar plate.

### Whole-genome sequencing, assembly, and genomic characterization of bacteriophages

Raw sequencing reads obtained from the sequencing platform were pre-processed using Fastp [66] to generate high-quality data for downstream analyses. SPAdes v3.15.5 was employed to assemble the high-quality reads into contigs. The completeness and quality of the assembled scaffolds were evaluated using CheckV v1.2.0 with default settings [67]. Phage taxonomy prediction was performed using PhaGCN (https://phage.ee.cityu.edu.hk/result-search), and the lytic or temperate nature of the phages was predicted using PhaTYP [68]. Gene prediction within the assembled phage genomes was conducted using Prokka v1.14.6 [69], followed by manual curation using NCBI BLAST against the non-redundant protein sequence database. The virulence factors and antibiotic resistance genes within the phage genomes were annotated using VFDB and the CARD database. All the raw sequencing data in this study have been uploaded to the NCBI database with the accession number PRJNA1313127, and the phage assembly data have been uploaded to the GenBank database with the accession numbers SAMN50885022-SAMN50885077.

### Transmission electron microscopy of phage morphology

Phage morphology was examined using negative staining with phosphotungstic acid. Briefly, 20 µL of concentrated and purified ultracentrifuged phage suspension was applied to a carbon-coated copper grid. After 5 minutes, the excess phage suspension was carefully removed with filter paper. The grid was then negatively stained with 5 µL of 2% (w/v) phosphotungstic acid for 2 minutes. Following air-drying, phage morphology was visualized and photographed using a transmission electron microscope (TEM).

### One-step growth curves

One-step growth curves, encompassing the latent, lysis, and plateau phases, are employed to delineate the bacteriophage life cycle. Bacteriophages were mixed with *K. pneumoniae* at the optimal multiplicity of infection (MOI) and allowed to adsorb at 37°C. The mixture was centrifuged at 5,000 × g for 5 minutes to remove unadsorbed phages from the supernatant. The pellet was then washed 2–3 times with LB medium, resuspended in 5 mL of LB medium, and incubated at 37°C with shaking at 220 rpm for 2 hours. Samples were collected at 20-minute intervals during the incubation period, and phage titers were determined using the double-layer agar plate method. The burst size was calculated based on the one-step growth curve, as previously described [70]. The one-step growth curve was plotted with time as the x-axis and the logarithm (base 10) of the phage titer as the y-axis, allowing for the determination of the latent period, lysis period, and burst size. The burst size was calculated as follows:

Burst size = Phage titer at plateau phase / Host concentration at initial infection.

### pH stability assay

To assess the pH stability of the bacteriophage, its titers were determined under various pH conditions. The pH of the SM buffer was adjusted to values ranging from 2 to 12 using HCl and NaOH. Subsequently, 900 μL of SM buffer at each pH value was mixed with 100 μL of phage solution and incubated at 37°C in a water bath for 1 hour. Immediately following incubation, samples were serially diluted, and phage titers were determined using the double-layer agar overlay method. The experiment was performed in triplicate, and a graph was generated with the logarithm (base 10) of the titer as the ordinate (y-axis) and pH as the abscissa (x-axis).

### Thermal stability assay

To assess the thermal stability of the bacteriophage, its titers were measured across a range of temperatures. Phage solutions were incubated for 1 hour at −80°C, −20°C, 4°C, 37°C, 50°C, 60°C, and 80°C. Phage titers were determined using the double-layer agar overlay method, with each temperature point performed in triplicate. The logarithm (base 10) of the phage titer was plotted against temperature.

### Phage genome editing

The phage genome editing procedure and recombinant plasmid construction were performed with modifications to the method described by Yu Wang et al. [71].

For the selection of phage mutants, a 1 mL fresh single colony overnight culture of *K. pneumoniae* carrying pCasKP and pSGKP-sgRNA plasmids was diluted into 100 mL of LB broth containing 30 µg/mL apramycin and 50 µg/mL kanamycin, and incubated at 30°C. When the cell density reached an OD_600 _ of approximately 0.2, 100 µL of the bacterial culture was mixed with 100 µL of wild-type phage vB_KP_P4 at various dilutions. The mixture was then added to 5 mL of 55°C LB semi-solid agar, gently inverted to mix, and poured onto pre-prepared LB solid agar plates. After drying at room temperature, the plates were incubated overnight at 30°C. Single plaques were picked, and phage mutants were screened by PCR amplification.

### In vitro bacteriophage inhibition assay

*K. pneumoniae* cells were cultured to the logarithmic growth phase. Aliquots of 100 µL of *K. pneumoniae* suspension (1 × 10^8^ CFU/mL) were mixed with equal volumes of bacteriophage at multiplicity of infection (MOI) ratios of 10, 1, 0.1, 0.01, and 0.001 in 96-well plates. The negative control consisted of 100 µL PBS and 100 µL LB broth, while the positive control comprised 100 µL *K. pneumoniae* suspension and 100 µL LB broth. Following the addition of all components, the 96-well plates were incubated at 37°C for 24 hours in an automated growth curve analyzer. Optical density at 600 nm (OD_600 _) was measured every 1 hour. The experiment was performed in triplicate, and the average values were calculated.

### Animal experiments

All BALB/c mice were individually identified and randomly allocated to the control and treatment groups using a random number generator. Twenty BALB/c mice were divided into two groups (*n* = 10 per group). All mice were intraperitoneally injected with 100 μL of *K. pneumoniae* bacterial suspension (2 × 10^9^ CFU/mL). One hour post-infection, the treatment groups (*n* = 10 per group) received intraperitoneal injections of 100 μL of phage suspension at a multiplicity of infection (MOI) of 10; the control group was established under identical conditions, receiving sterile PBS in place of the phage. Clinical symptoms and survival rates were monitored for seven consecutive days. Yueren Xu knew the group allocation for the survival rate experiment.

Ten BALB/c mice were divided into two groups (*n* = 5 per group). The bacterial suspension was prepared and adjusted to a titer of 2 × 10^9^ CFU/mL. Subsequently, 100 μL of this bacterial suspension was administered to mice via intraperitoneal injection for infection challenge. One hour post-infection, mice were treated with bacteriophage at a titer of 2 × 10^10^ PFU/mL, also administered via intraperitoneal injection (*n* = 5). A control group was established under identical conditions, receiving sterile PBS in place of the phage (*n* = 5). The liver, spleen, kidney, and lung tissues were collected after 12 hours post-phage treatment, and bacterial burdens were quantified for comparative analysis. Keyu Zhu knew about the group allocation for this experiment.

To minimize errors, the personnel evaluating the results were unaware of the animals’ treatment status. The cages were randomly positioned and periodically changed. The sample size for this study was determined based on literature related to research on *K. pneumoniae* phage therapy [72] and the desired statistical power of 80% at α = 0.05. Bacterial load (log_10_ CFU/g) was the primary endpoint, where *n* = 5 is statistically sufficient to detect the anticipated large effect size (2–5 log 10 reduction). *n* = 10 was used for the survival study as the minimal, acceptable standard for robust Log-Rank analysis.

## Statistical analysis

Statistical analyses were conducted using GraphPad Prism 8.0.2 and the R statistical. Group differences were assessed via the Student’s t-test. Prior to this, data assumptions, including normality and homogeneity of variances; non-parametric tests would have been employed if assumptions were violated. For the survival study, compared using the Log-Rank (Mantel-Cox) test. Line graphs and bar charts were generated using GraphPad Prism and the ggplot2 package in R. *p*-value <0.05 (*) was considered statistically significant. (**): *p* < 0.01, (***): *p *< 0.001.

## Supplementary Material

Clean252121694_Revised_supplementary_figures.docx

252121694_Revised supplementary tables.xls

Author Checklist E10 only.pdf

## Data Availability

The data that support the findings of this study are openly available in Figshare at https://doi.org/10.6084/m9.figshare.30577973 [73]. The raw sequence data have been uploaded to the NCBI database with the BioProject number PRJNA1313127, and the phage assembly data have been uploaded to the GenBank database with the BioSample numbers SAMN50885022–SAMN50885077 [74].
