# Supplementary material for: Phage anti-defense genes targeting NAD+ metabolism and DNA replication are associated with a broad host range against Klebsiella pneumoniae
Source: Virulence. 2026 Aug 24;17(1):2709261. doi: 10.1080/21505594.2026.2709261 (PMC13505451; doi:10.1080/21505594.2026.2709261)
Supplement: Supplementary file 2 [file KVIR_A_2709261_SM5288.docx]

Supplementary Figure S1. Lytic Profiles of 56 Phage Isolates. An unrooted phylogenetic tree of the phages, depicting 56 phages, comprising 6 temperate phages and 50 lytic phages.

Supplementary Figure S2. Defense Systems Identified in *K. pneumoniae* Isolates. Defense systems identified in *K. pneumoniae* isolates. The number of instances of each defense system type per strain is represented by red, orange, or white, indicating 2, 1, or 0, respectively. A comprehensive list of defense systems identified in the *K. pneumoniae* isolates can be found in Supplementary Table S6.

### Supplementary Figure S3. Genomic contexts and predicted independent promoters of host-range determinant genes. (A) *comEB* (ORF_035) is driven by promoter P1 (LDF = 1.75) with a 16 bp intergenic gap from the downstream ORF. (B) *nadM* (ORF_091) possesses a robust promoter P3 (LDF = 6.54) located within a 174 bp upstream region, followed by a 176 bp non-coding spacer. (C) *rfcS* (ORF_273) is antisense-transcribed via promoter P4 (LDF = 1.13) within a 152bp regulatory window. Promoters were predicted using BPROM. Arrows and bent arrows indicate transcription direction and start sites, respectively.

Supplementary Figure S4. Genomic hallmarks of broad-host-range *K. pneumoniae* phages. (A) Genomic distribution of the three target genes across phages with broad and narrow host ranges. (B) Phylogenetic analysis based on whole-genome sequences, highlighting the evolutionary relationships between vB_KP_P4 (red) and clinically potent phages such as phiKp8 and phiKp26. (C–E) Comparative genomic analysis of the core host-range determinants: *comEB* (C), *nadM* (D), and *rfcS* (E). The colored arrows represent ORFs, with red arrows indicating the target genes. Gray shaded areas between genomes denote nucleotide identity levels (98%–100%), illustrating the high degree of structural synteny and sequence conservation of these anti-defense modules across different phage backgrounds.
